# Human receptor kinetics and lung tissue retention of the enhanced-affinity glucocorticoid fluticasone furoate

**DOI:** 10.1186/1465-9921-8-54

**Published:** 2007-07-25

**Authors:** Anagnostis Valotis, Petra Högger

**Affiliations:** 1 Universität Würzburg, Institut für Pharmazie und Lebensmittelchemie, Würzburg, Germany

## Abstract

Fluticasone furoate (FF) – USAN approved name, a new topically active glucocorticoid has been recently identified. The aim of this study was to characterise the binding affinity of this compound to the human lung glucocorticoid receptor in relation to other glucocorticoids. Additionally, we sought to determine the binding behaviour of fluticasone furoate to human lung tissue. The glucocorticoid receptor binding kinetics of fluticasone furoate revealed a remarkably fast association and a slow dissociation resulting in a relative receptor affinity (RRA) of 2989 ± 135 with reference to dexamethasone (RRA: 100 ± 5). Thus, the RRA of FF exceeds the RRAs of all currently clinically used corticosteroids such as mometasone furoate (MF; RRA 2244), fluticasone propionate (FP; RRA 1775), ciclesonide's active metabolite (RRA 1212 – *rat *receptor data) or budesonide (RRA 855). FP and FF displayed pronounced retention in human lung tissue *in vitro*. Lowest tissue binding was found for MF. There was no indication of instability or chemical modification of FF in human lung tissue. These advantageous binding attributes may contribute to a highly efficacious profile for FF as a topical treatment for inflammatory disorders of the respiratory tract.

## Background

**Figure 1:**
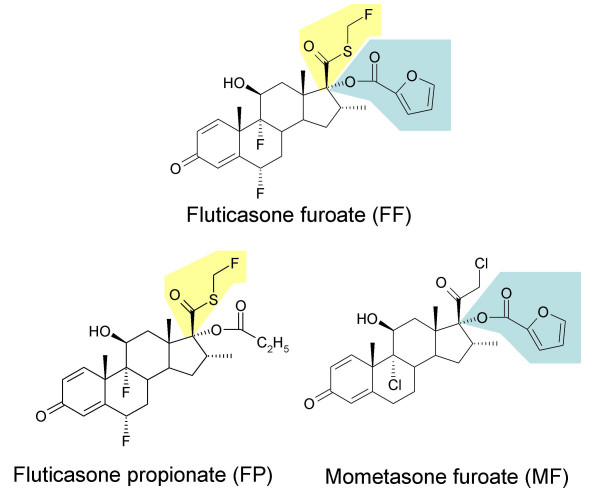
Structural formulae of the new glucocorticoid fluticasone furoate in comparison with fluticasone propionate and mometasone furoate.

A new topically active glucocorticoid, fluticasone furoate (FF, GW685698X), has been recently identified (Figure 1) and is being progressed for the treatment of respiratory diseases. Fluticasone furoate (FF) shares structural similarities with fluticasone propionate (FP) with the exception of the substitution of the 17-α hydroxyl group. While this position is esterified with propionic acid in FP, FF carries a 2-furoate ester moiety.

For topically applied glucocorticoids, it is favorable to combine high local efficacy with low systemic exposure. An enhanced affinity for lung tissue may prolong residence time in the lung and minimise systemic effects. Therefore, a high receptor affinity and a high retention in the target tissue should be paralleled by rapid and complete hepatic metabolism of the glucocorticoid to inactive derivatives. We previously described the receptor binding affinity of FP and MF as well as their retention in lung tissue *in vitro *[1-4]. Both FP and MF have high affinities for the human lung glucocorticoid receptor. The relative receptor affinity (RRA) of FP is about 1800 compared to the reference compound dexamethasone (RRA= 100), the RRA of MF is about 2250.

The aim of this study was to characterise the binding affinity of the novel compound FF to the glucocorticoid receptor in relation to other glucocorticoids. Therefore, we isolated human lung glucocorticoid receptors from human lung tissue and determined the binding affinity of these glucocorticoids by the kinetic method described earlier [1]. Additionally, we sought to determine the retention of FF in human lung tissue.

## Methods

### Chemicals and reagents

[^3^H]-Dexamethasone was obtained from Amersham (Freiburg, Germany), dexamethasone was purchased from Merck (Darmstadt, Germany). [^3^H]-Fluticasone furoate, FP, MF, FF, ciclesonide (Cicle) and its active metabolite desisobutyryl-ciclesonide (des-Cicle), beclomethasone-17, 21-dipropionate (BDP) and its metabolite beclomethasone-17-monopropionate (17-BMP) and beclomethasone-21-monopropionate (21-BMP) were generous gifts from GlaxoSmithKline (Greenford, England). The origin of all other glucocorticoids mentioned is described in [5]. Dimethyl-2-2-dichlorvinylphosphate (dichlorvos) was purchased from Riedel de Haën (Seelze, Germany), DL-dithiothreitol (DTT) from Sigma-Aldrich-Chemie (Taufkirchen, Germany). Complete™ (combination of different protease inhibitors) was obtained from Roche Applied Science (Mannheim, Germany), Norit A from Serva (Heidelberg, Germany). Diethylether (HPLC grade) was purchased from Fluka (Buchs, Switzerland) and acetonitrile (ACN, HPLC gradient grade) from Fisher Scientific, (Schwerte, Germany). Water from a Millipore water purification unit was used. All other chemicals were obtained from E. Merck (Darmstadt, Germany).

### Buffer solutions

Buffer solution G contained 10 mM TRIS, 10 mM Na_2_MoO_4_, 30 mM NaCl, 10 % glycerol (pH 7.4). Buffer solution A contained 4 mM DTT, 5 mM dichlorvos and 1 mM Complete™ in 100 mL buffer solution G. Krebs-Ringer-HEPES buffer (pH 7.4) consisted of 118 mM NaCl, 4.84 mM KCl, 1.2 mM KH_2_PO_4_, 2.43 mM MgSO_4_, 2.44 mM CaCl_2 _× 2 H_2_O and 10 mM HEPES.

### Source and handling of human specimen

Human lung tissue resection material was obtained from patients with bronchial carcinomas who gave informed consent. Cancer-free tissue was used for the experiments. None of the patients was treated with glucocorticoids for the last 4 weeks prior to surgery. Tissue samples were used immediately for tissue metabolism studies to retain full enzymatic activity. For other experiments, tissue samples were shock frozen in liquid nitrogen after resection and stored at -70°C until usage. To collect sufficient material for the experiments tissue samples of three or more patients were pooled.

Plasma samples were obtained from healthy volunteers who gave informed consent. Samples were used immediately for metabolism studies to retain full enzymatic activity. For desorption and other experiments, plasma samples were shock frozen in liquid nitrogen and stored at -70°C until usage.

### Preparation of lung cytosol for receptor binding experiments

Human lung tissue was deep frozen immediately after resection and stored in liquid nitrogen. Frozen tissue was pulverized and homogenized in three aliquots buffer solution A with an Ultra Turrax mixer (Janke and Kunkel, Staufen, Germany) in an ice bath. Thereafter the diluted cytosol was centrifuged for 1 hr at 105,000 × g at 4°C (Ultracentrifuge L8-55 M, Beckman Instruments Irvine, California). The cytosol was stored in aliquots at -70°C. The protein concentration of the cytosol was determined according to the method of Lowry et al. [6]. Concentration of glucocorticoid receptors in the cytosol was 30–60 fmol/mg protein.

### Kinetics of receptor binding of glucocortiocids

The receptor binding experiments were performed according to the procedure described earlier [1] based on [7-9].

#### A. Determination of receptor number in the cytosol and calculation of equilibrium dissociation rate constant

Various dilutions of [^3^H]-dexamethasone in buffer solution G (6 × 10^-7 ^to 1.2 × 10^-8 ^mol/L) were prepared. For elucidation of non-specific binding a solution of dexamethasone (1.2 × 10^-5 ^mol/L) in buffer solution G was used. For the assay of non-specific binding (B_ns _in [mol/L]), 20 μL of [^3^H]-dexamethasone and 20 μL of the unlabelled compound were added to 200 μL of cytosol, were mixed in glass vials and incubated for 18 to 20 h at 0–4°C. The assay for total binding (B_t _in [mol/L]) was carried out accordingly, but the unlabelled glucocorticoid was replaced by buffer solution G. To determine the total [^3^H]-glucocorticoid concentration (T), 20 μL of the mixture were used for scintillation counting. After incubation, 200 μL of each incubation mixture were added to 200 μL suspension of activated charcoal (2 % Norit A in buffer solution G), incubated for 10 min on ice and centrifuged for 5 min between 0–4°C. For scintillation counting 200 μL of the supernatant were used. Scintillation counting was performed with a Rackbeta 1214 LKB from Wallac (Freiburg, Germany) using Emulsifier-Safe™ from Packard Bioscience (Groningen, Netherlands).

Receptor concentration (R_0_) of the cytosol was calculated by the method of Scatchard [10] according the equation:

[Bs][H]=[R0]KD−[Bs]KD
 MathType@MTEF@5@5@+=feaafiart1ev1aaatCvAUfKttLearuWrP9MDH5MBPbIqV92AaeXatLxBI9gBaebbnrfifHhDYfgasaacH8akY=wiFfYdH8Gipec8Eeeu0xXdbba9frFj0=OqFfea0dXdd9vqai=hGuQ8kuc9pgc9s8qqaq=dirpe0xb9q8qiLsFr0=vr0=vr0dc8meaabaqaciaacaGaaeqabaqabeGadaaakeaadaWcaaqaamaadmaabaGaemOqai0aaSbaaSqaaiabdohaZbqabaaakiaawUfacaGLDbaaaeaadaWadaqaaiabdIeaibGaay5waiaaw2faaaaacqGH9aqpdaWcaaqaamaadmaabaGaemOuai1aaSbaaSqaaiabicdaWaqabaaakiaawUfacaGLDbaaaeaacqWGlbWsdaWgaaWcbaGaemiraqeabeaaaaGccqGHsisldaWcaaqaamaadmaabaGaemOqai0aaSbaaSqaaiabdohaZbqabaaakiaawUfacaGLDbaaaeaacqWGlbWsdaWgaaWcbaGaemiraqeabeaaaaaaaa@4427@

with B_S _being the specific binding of the labelled dexamethasone in [mol/L], H being the unbound labelled glucocorticoid, and K_D _being the equilibrium dissociation rate constant. B_S _and H were indirectly determined using the equations:

[*B*_*s*_] = [*B*_*t*_] - [*B*_*ns*_]

[*H*] = [*T*] - [*B*_*t*_]

The Scatchard plot revealed the equilibrium dissociation rate constant K_D _(slope of the straight line) and the receptor number R_0 _in mol receptors per mg total protein of the cytosol (interception of the straight line with the x-axis).

#### B. Determination of association rate constants k_Ass _(= k_1_)

For the determination of the association rate constant, the cytosol was incubated with different concentrations of [^3^H]-glucocorticoid in the absence and presence of excess unlabelled glucocorticoid. For the assay of non-specific binding, 10 parts of cytosol, 1 volume part of [^3^H]-glucocorticoid (1.2 × 10^-7 ^mol/L) and 1 volume part of cold glucocorticoid (1.2 × 10^-4 ^mol/L) were mixed in glass vials and incubated at 20°C. The assay for total binding was carried out accordingly, but the unlabelled glucocorticoid (1.2 × 10^-4 ^mol/L) was replaced by buffer G. To determine the total [^3^H]-glucocorticoid concentration, aliquots of the incubation mixtures were used for scintillation counting. At intervals, 200 μL incubation mixture were mixed with 200 μL suspension of Norit A, incubated for 10 min on ice and centrifuged for 5 min between 0–4°C. For scintillation counting 200 μL of the supernatant were used.

The association rate constant (k_Ass _= k_1_) of the cytosol was calculated according the equation:

Zt=ln⁡([Gt]/[Rt])[G0]−[R0]=KASS⋅t+ln⁡([G0]/[R0])[G0]−[R0]
 MathType@MTEF@5@5@+=feaafiart1ev1aaatCvAUfKttLearuWrP9MDH5MBPbIqV92AaeXatLxBI9gBaebbnrfifHhDYfgasaacH8akY=wiFfYdH8Gipec8Eeeu0xXdbba9frFj0=OqFfea0dXdd9vqai=hGuQ8kuc9pgc9s8qqaq=dirpe0xb9q8qiLsFr0=vr0=vr0dc8meaabaqaciaacaGaaeqabaqabeGadaaakeaacqWGAbGwdaWgaaWcbaGaemiDaqhabeaakiabg2da9maalaaabaGagiiBaWMaeiOBa42aaeWaaeaadaWadaqaaiabdEeahnaaBaaaleaacqWG0baDaeqaaaGccaGLBbGaayzxaaGaei4la8YaamWaaeaacqWGsbGudaWgaaWcbaGaemiDaqhabeaaaOGaay5waiaaw2faaaGaayjkaiaawMcaaaqaamaadmaabaGaem4raC0aaSbaaSqaaiabicdaWaqabaaakiaawUfacaGLDbaacqGHsisldaWadaqaaiabdkfasnaaBaaaleaacqaIWaamaeqaaaGccaGLBbGaayzxaaaaaiabg2da9iabdUealnaaBaaaleaacqWGbbqqcqWGtbWucqWGtbWuaeqaaOGaeyyXICTaemiDaqNaey4kaSYaaSaaaeaacyGGSbaBcqGGUbGBdaqadaqaamaadmaabaGaem4raC0aaSbaaSqaaiabicdaWaqabaaakiaawUfacaGLDbaacqGGVaWldaWadaqaaiabdkfasnaaBaaaleaacqaIWaamaeqaaaGccaGLBbGaayzxaaaacaGLOaGaayzkaaaabaWaamWaaeaacqWGhbWrdaWgaaWcbaGaeGimaadabeaaaOGaay5waiaaw2faaiabgkHiTmaadmaabaGaemOuai1aaSbaaSqaaiabicdaWaqabaaakiaawUfacaGLDbaaaaaaaa@6A21@

with G_t _being the concentration of unbound labelled glucocorticoid at time t, R_t _being the concentration of free receptors at time t, G_0 _being the concentration of unbound labelled glucocorticoid at time t = 0, R_0 _being the concentration of free receptors at time t = 0 and t being the time of incubation. G_0 _and G_t _were indirectly determined using the equations:

[*G*_0_] = [*T*] - [*B*_*ns*,0_] and [*G*_*t*_] = [*T*] - [*B*_*ns*,*t*_]

To linearize the calculated data points a Z_t_-value was calculated for each time point of measurement taking the dilution factor of the cytosol and the receptor concentration into account:

Zt=ln⁡{([T]−[Bns,t])/([R0]−[BT,t]+[Bns,t])}[T]−[Bns,t]−[R0]
 MathType@MTEF@5@5@+=feaafiart1ev1aaatCvAUfKttLearuWrP9MDH5MBPbIqV92AaeXatLxBI9gBaebbnrfifHhDYfgasaacH8akY=wiFfYdH8Gipec8Eeeu0xXdbba9frFj0=OqFfea0dXdd9vqai=hGuQ8kuc9pgc9s8qqaq=dirpe0xb9q8qiLsFr0=vr0=vr0dc8meaabaqaciaacaGaaeqabaqabeGadaaakeaacqWGAbGwdaWgaaWcbaGaemiDaqhabeaakiabg2da9maalaaabaGagiiBaWMaeiOBa42aaiWabeaadaqadaqaamaadmaabaGaemivaqfacaGLBbGaayzxaaGaeyOeI0YaamWaaeaacqWGcbGqdaWgaaWcbaGaemOBa4Maem4CamNaeiilaWIaemiDaqhabeaaaOGaay5waiaaw2faaaGaayjkaiaawMcaaiabc+caVmaabmaabaWaamWaaeaacqWGsbGudaWgaaWcbaGaeGimaadabeaaaOGaay5waiaaw2faaiabgkHiTmaadmaabaGaemOqai0aaSbaaSqaaiabdsfaujabcYcaSiabdsha0bqabaaakiaawUfacaGLDbaacqGHRaWkdaWadaqaaiabdkeacnaaBaaaleaacqWGUbGBcqWGZbWCcqGGSaalcqWG0baDaeqaaaGccaGLBbGaayzxaaaacaGLOaGaayzkaaaacaGL7bGaayzFaaaabaWaamWaaeaacqWGubavaiaawUfacaGLDbaacqGHsisldaWadaqaaiabdkeacnaaBaaaleaacqWGUbGBcqWGZbWCcqGGSaalcqWG0baDaeqaaaGccaGLBbGaayzxaaGaeyOeI0YaamWaaeaacqWGsbGudaWgaaWcbaGaeGimaadabeaaaOGaay5waiaaw2faaaaaaaa@6CBD@

The Z_t_-values were plotted against time t and a linear regression was performed. The slope of the straight line (k_Ass _= k_1_) and the coefficient of correlation r were calculated based on a minimum of four data points. The coefficient of correlation was always higher than r = 0.975.

#### C. Determination of dissociation rate constants k_Diss_(= k_-1_)

For determination of the dissociation rate constant, 10 volume parts cytosol and 1 volume part [^3^H]-glucocorticoid solution (6 × 10^-7 ^mol/L) were incubated for 18–20 h between 0–4°C (mixture 1). To determine the non-specific binding, 10 volume parts of cytosol, 1 volume part of [^3^H]-glucocorticoid solution (6 × 10^-7 ^mol/L) and 1 part of unlabelled glucocorticoid (3 × 10^-4 ^mol/L) were incubated for 18–20 h between 0–4°C (mixture 2). Incubation mixtures were subsequently brought to a temperature of 20°C. One volume part of unlabelled glucocorticoid (3 × 10^-4 ^mol/L) was added to mixture 1. At intervals 200 μL each of the mixtures 1 and 2 were mixed with 200 μL Norit A suspension, incubated at 0–4°C for 10 min and thereafter centrifuged for 5 min at 0–4°C. The supernatant was used for scintillation counting. The first order rate constant was calculated according:

[Bs,t]=[Bs,0]⋅e−KDiss⋅t
 MathType@MTEF@5@5@+=feaafiart1ev1aaatCvAUfKttLearuWrP9MDH5MBPbIqV92AaeXatLxBI9gBaebbnrfifHhDYfgasaacH8akY=wiFfYdH8Gipec8Eeeu0xXdbba9frFj0=OqFfea0dXdd9vqai=hGuQ8kuc9pgc9s8qqaq=dirpe0xb9q8qiLsFr0=vr0=vr0dc8meaabaqaciaacaGaaeqabaqabeGadaaakeaadaWadaqaaiabdkeacnaaBaaaleaacqWGZbWCcqWGSaalcqWG0baDaeqaaaGccaGLBbGaayzxaaGaeyypa0ZaamWaaeaacqWGcbGqdaWgaaWcbaGaem4CamNaemilaWIaeGimaadabeaaaOGaay5waiaaw2faaiabgwSixlabdwgaLnaaCaaaleqabaGaeyOeI0Iaem4saS0aaSbaaWqaaiabdseaejabdMgaPjabdohaZjabdohaZbqabaWccqGHflY1cqWG0baDaaaaaa@4A2E@

with B_s,t _being the specific binding of the labelled compound at time t, B_s,0 _being the specific binding of the labelled compound at time t = 0. Since the specific binding was determined indirectly (see *A*) the equation can be rewritten as:

[BT,t]−[Bns,t]=([BT,0]−[Bns,t])⋅e−KDiss⋅t
 MathType@MTEF@5@5@+=feaafiart1ev1aaatCvAUfKttLearuWrP9MDH5MBPbIqV92AaeXatLxBI9gBaebbnrfifHhDYfgasaacH8akY=wiFfYdH8Gipec8Eeeu0xXdbba9frFj0=OqFfea0dXdd9vqai=hGuQ8kuc9pgc9s8qqaq=dirpe0xb9q8qiLsFr0=vr0=vr0dc8meaabaqaciaacaGaaeqabaqabeGadaaakeaadaWadaqaaiabdkeacnaaBaaaleaacqWGubavcqGGSaalcqWG0baDaeqaaaGccaGLBbGaayzxaaGaeyOeI0YaamWaaeaacqWGcbGqdaWgaaWcbaGaemOBa4Maem4CamNaeiilaWIaemiDaqhabeaaaOGaay5waiaaw2faaiabg2da9maabmaabaWaamWaaeaacqWGcbGqdaWgaaWcbaGaemivaqLaeiilaWIaeGimaadabeaaaOGaay5waiaaw2faaiabgkHiTmaadmaabaGaemOqai0aaSbaaSqaaiabd6gaUjabdohaZjabcYcaSiabdsha0bqabaaakiaawUfacaGLDbaaaiaawIcacaGLPaaacqGHflY1cqWGLbqzdaahaaWcbeqaaiabgkHiTiabdUealnaaBaaameaacqWGebarcqWGPbqAcqWGZbWCcqWGZbWCaeqaaSGaeyyXICTaemiDaqhaaaaa@5DC7@

The B_s,t_-values were plotted semi-logarithmical against time t and a linear regression was performed. The slope of the straight line (k_Diss _= k_-1_) and the coefficient of correlation r were calculated based on a minimum of six data points. The coefficient of correlation was always higher than r = 0.975.

Equilibrium dissociation constant (K_D_) was calculated for each glucocorticoid based on association and dissociation rate constants:

KD=k−1k1
 MathType@MTEF@5@5@+=feaafiart1ev1aaatCvAUfKttLearuWrP9MDH5MBPbIqV92AaeXatLxBI9gBaebbnrfifHhDYfgasaacH8akY=wiFfYdH8Gipec8Eeeu0xXdbba9frFj0=OqFfea0dXdd9vqai=hGuQ8kuc9pgc9s8qqaq=dirpe0xb9q8qiLsFr0=vr0=vr0dc8meaabaqaciaacaGaaeqabaqabeGadaaakeaacqWGlbWsdaWgaaWcbaGaemiraqeabeaakiabg2da9maalaaabaGaem4AaS2aaSbaaSqaaiabgkHiTiabbgdaXaqabaaakeaacqWGRbWAdaWgaaWcbaGaeeymaedabeaaaaaaaa@3607@

Relative receptor affinities (RRA) for glucocorticoids (GC) were calculated with reference to dexamethasone (Dexa):

RRA=KD DexaKD GC×100
 MathType@MTEF@5@5@+=feaafiart1ev1aaatCvAUfKttLearuWrP9MDH5MBPbIqV92AaeXatLxBI9gBaebbnrfifHhDYfgasaacH8akY=wiFfYdH8Gipec8Eeeu0xXdbba9frFj0=OqFfea0dXdd9vqai=hGuQ8kuc9pgc9s8qqaq=dirpe0xb9q8qiLsFr0=vr0=vr0dc8meaabaqaciaacaGaaeqabaqabeGadaaakeaacqWGsbGucqWGsbGucqWGbbqqcqGH9aqpdaWcaaqaaiabdUealnaaBaaaleaacqWGebaraeqaaOGaeeiiaaIaemiraqKaemyzauMaemiEaGNaemyyaegabaGaem4saS0aaSbaaSqaaiabdseaebqabaGccqqGGaaicqWGhbWrcqWGdbWqaaGaey41aqRaeeymaeJaeeimaaJaeeimaadaaa@439D@

### Stability of fluticasone furoate (FF) in fresh human lung tissue in vitro

Fluticasone furoate (FF) (0.3 μg/mL) was incubated in 10 ml Krebs-Ringer-HEPES buffer with lung tissue pieces at 37°C shielded from light in a thermostatically controlled shaking water bath GFL 1083 (Burgwedel, Germany). Incubations were performed in the presence and absence of dichlorvos (1 mg/mL). Over 24 hours, samples of 1.0 mL tissue-free supernatant were taken and immediately stored at -20°C until analysis. The incubation medium was replenished by buffer which was pre-temperated to 37°C. In case of incubations with dichlorvos the medium used for replenishment contained the esterase inhibitor.

### Adsorption of glucocorticoids to lung tissue

Lung tissue was washed in Krebs-Ringer-HEPES buffer (pH 7.4) and sliced into pieces of 1 mm^3^. For each binding experiment approximately 0.5 g of lung tissue was used. Adsorption of glucocorticoids (0.3 μg/mL) to human lung tissue was determined as described earlier [3]. Briefly, lung tissue pieces were suspended under gentle shaking for 1 h at 37°C in 20 ml Krebs-Ringer-HEPES buffer containing 0.3 μg/ml of the glucocorticoid. 2.0 mL samples were taken and stored at -20°C until analysis. The volume withdrawn was replaced with fresh buffer of 37°C. Only glass lab ware was used for these experiments to avoid any non-specific binding effects of the highly lipophilic compounds to plastic material. For control, blank samples with glucocorticoid-containing buffer, but no tissue, were incubated under the same experimental conditions (1 h at 37°C, in Krebs-Ringer-HEPES buffer) and analyzed for non-specific adsorption of the glucocorticoids to the glass tubes.

### Desorption of glucocorticoids from lung tissue

Desorption of glucocorticoids to human lung tissue was determined as described earlier [3]. Briefly, lung tissue (1.0 g) was saturated with glucocorticoids for 1 h at 37°C by shaking in 40 mL Krebs-Ringer-HEPES buffer containing 0.3 μg/mL of the respective glucocorticoid. After incubation tissue was washed with 2 mL buffer and transferred into 10.0 mL human plasma (37°C). Again, only glass lab ware was used for these experiments to exclude any non-specific binding effects of the highly lipophilic compounds to plastic material. Samples of 1.0 mL were taken at defined time points. The volume was replaced with fresh plasma at 37°C. Samples were stored at -20°C until further analysis.

### Sample preparation, HPLC conditions and data analysis

Samples of 1.0 mL (tissue desorption/stability) or 2.0 mL (tissue adsorption) were mixed with 0.1 mL internal standard solution and extracted twice with 3 mL diethylether for 30 min, using a roller mixer, followed by centrifugation (20°C) for 5 min. The organic phase was separated and evaporated to dryness under a gentle stream of nitrogen at 25°C. The resulting residue was reconstituted in 0.2 mL mobile phase. Internal standard (IS) was amcinonide 3 μg/mL (tissue binding studies) or dexamethasone 3 μg/mL (stability studies). Linearity was given from 10–500 ng/mL glucocorticoid, coefficients of correlation of the calibration curves were at least 0.99.

The HPLC system was a Waters HPLC (Milford, MA) consisting of a 1525 binary pump, an 717plus autosampler and 2487 dual wavelength absorbance detector set at the detection wavelength of 254 nm. Data collection and integration were accomplished using Breeze™ software version 3.2. Analysis was performed on a Symmetry C_18 _column (150 × 4.6 mm I.D., 5 μm particle size, Waters, MA). Typically, 20 μL of sample were injected and separated at a flow rate of 1 mL/min. Gradient elution was performed using water (containing 0.2 % (v/v) acetic acid) and ACN, starting at 60:40 (v/v) water/ACN increasing linearly to 29:71 (v/v) water/ACN by 30 min. The assay was accurate and reproducible. The lower limit of quantitation was 10 ng/mL for all glucocorticoids except ciclesonide (20 ng/mL).

### Determination of the relative retention time k' of glucocorticoids

Relative retention times k' or chromatographic capacity factors log (k'), respectively, of all new generation glucocorticoids in comparison with older glucocorticoids were determined by a HPLC method based on a former report [5]. Briefly, to calculate k' the HPLC retention time on a C_18 _reversed-phase column of an individual glucocorticoid was related to the retention time of an internal standard (dexamethasone-21-isonicotinate). Therefore, 10 μL of the respective glucococorticoid and the internal standard at a concentration of each 10 μg/mL in methanol were chromatographed under identical conditions (column and HPLC system described above). The sample was injected and separated at a flow rate of 0.7 mL/min. The mobile phase consisted of methanol, water, ACN and acetic acid at 40:20:5:0.2 (v/v).

### Statistical analysis

Mean and mean deviation of the mean were calculated for all data. Data sets were analysed by one-way ANOVA with post-hoc Bonferroni's multiple comparison test. Statistical significance was defined as a significance level of p ≤ 0.05. Due to the very limited sample number a pre-test was performed to test the normal distribution of the residuals. Therefore, the residuals of each data group were calculated and the ratio of range to standard deviation was analysed according to David et al. [11]. Only when the results were between the lower and upper critical limits tabulated by Pearson and Stephens [12] a normal distribution of the residuals was assumed at a significance level of p ≤ 0.05 and a subsequent ANOVA analysis was performed. On one data set a reciprocal transformation was performed for normal distribution of the residuals and subsequent ANOVA analysis. Due to the limited number of data p values should be interpreted very cautiously.

## Results

### Receptor binding kinetics and relative receptor affinity of fluticasone furoate (FF)

The receptor binding kinetics to the human lung glucocorticoid receptor revealed that the association kinetics of fluticasone furoate (FF) was distinctly different from those of fluticasone propionate (FP) and mometasone furoate (MF) (Table 1). The association rate constant of FF was statistically significantly higher compared to both MF and FP (both p ≤ 0.001); thus the specific binding to the receptor occurred more rapidly and to a higher extent compared with all other glucocorticoids. In contrast, the dissociation rate constant of FF was comparable with that of FP and MF with no statistically significant difference. Consequently, the calculated half-lives of the glucocorticoid-receptor complexes (t_1/2_) of FF, FP and MF were all around 10 hours. Equilibrium dissociation rate constants (k_d_) were derived from the association and dissociation rate constants. The calculated k_d _of FF was 0.30 nmol/L, the lowest among the tested glucocorticoids (statistically significantly lower compared to FP, p ≤ 0.001, and to MF, p ≤ 0.05). The k_d _of FP was 0.51 nmol/L, the k_d _of MF was determined as 0.41 nmol/L (statistically significantly different, p ≤ 0.05). Based on the equilibrium dissociation rate constants the relative receptor affinity (RRA) of FF was calculated as 2989 ± 135. This RRA of FF was significantly higher compared to FP, p ≤ 0.001, and to MF, p ≤ 0.05.

**Table 1:** Results of the kinetic binding experiments of dexamethasone (Dexa), fluticasone furoate (FF), fluticasone propionate (FP) and mometasone furoate (MF) to the human lung glucocorticoid receptor. Values given represent mean and mean deviation of the mean of three to seven experiments. Binding data of FP and MF are from our previous experiments (Ref. [3]).

Glucocorticoid	k_1 _× 10^5 ^(L/[mol/min])	k_-1 _× 10^-4 ^[1/min]	K_D _[nmol/L]	t_1/2 _[h]	RRA
Dexa	10.53 ± 0.35	94.67 ± 5.43	8.80 ± 0.41	1.23 ± 0.04	100 ± 5
**FF**	**37.46 ± 0.73**	**11.22 ± 0.62**	**0.30 ± 0.02**	**10.34 ± 0.59**	**2989 ± 135**
FP	21.17 ± 0.56	10.73 ± 0.65	0.51 ± 0.03	10.82 ± 0.64	1775 ± 130
MF	29.46 ± 1.10	11.82 ± 0.31	0.41 ± 0.03	9.83 ± 0.53	2244 ± 142

Statistically significant differences were observed in the association rate constant k_1 _(FF *versus *FP p ≤ 0.001; FF *versus *MF p ≤ 0.001; FP *versus *MF p ≤ 0.001), in equilibrium dissociation rate constant k_D _(FF *versus *FP p ≤ 0.001; FF *versus *MF p ≤ 0.05; FP *versus *MF p ≤ 0.05) and in the relative receptor affinity RRA (FF *versus *FP p ≤ 0.001; FF *versus *MF p ≤ 0.05; FP *versus *MF p ≤ 0.01). No statistically significant difference between FF, MF and FP was seen in the dissociation rate constant k_-1 _and the derived half life of the receptor complex t_1/2_.

### Correlation between glucocorticoid lipophilicy and receptor affinity

**Figure 2:**
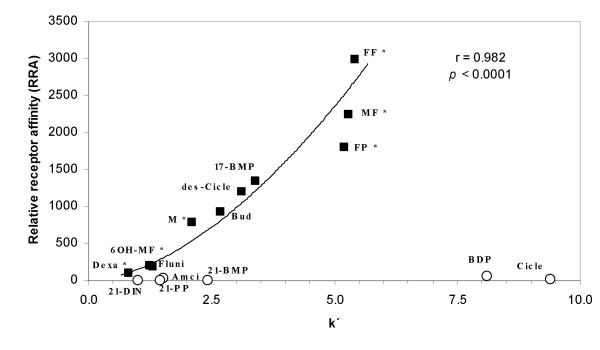
Relationship between the relative receptor affinities (RRA) of glucocorticoids and their lipophilicity expressed as relative retention times (k'). The reference glucocorticoid was dexamethasone for RRA and dexamethasone-21-isonicotinate for k'. Coefficient of correlation was r = 0.982 and the correlation was statistically significant (p ≤ 0.0001). Symbols used: *filled black squares*: glucocorticoids without ester function at C21 *open white circles*: glucocorticoids esterified at C21. * RRA determined in our own experiments, all other RRAs were obtained from [5, 16]. *Abbreviations*: Amcinonide (Amci), Dexamethasone (Dexa), Dexamethasone-21-isonicotinate (21-DIN), Flunisolide (Fluni), Fluticasone propionate (FP), Fluticasone furoate (FF), Mometasone (M), 6β-Hydroxy-Mometasone furoate (6OH-MF), Mometasone furoate (MF), Budesonide (Bud), Ciclesonide (Cicle), desisobutyryl Ciclesonide (des-Cicle), Beclomethasone-17,21-dipropionate (BDP), Beclomethasone-17-monopropionate (17-BMP), Beclomethasone-21-monopropionate (21-BMP), Prednisolone-21-propionate (21-PP).

The chromatographic capacity factor log (k') reveals an excellent correlation to the partition coefficient in 1-octanol-water [13,14] which is regarded as a typical parameter of compound lipophilicity. When the lipophilicity of a glucocorticoid is expressed as its relative retention time k' at a reversed-phase HPLC column and correlated with the relative receptor affinity of the respective compound, a significant relationship is observed (Figure 2). Potential fitting of the data according to the equation: y = c * x^b^. (with c and b representing constants) revealed a coefficient of correlation of r = 0.982. This relationship is statistically significant (p < 0.0001). All glucocorticoids esterified at C21 display higher lipophilicity. However, these compounds have little or no binding affinity to the glucocorticoid receptor. They are either inactive metabolites such as beclomethasone-21-monopropionate (21-BMP) or inactive pro-drugs such as ciclesonide or beclomethasone-17,21-dipropionate which need to be activated by hydrolysis of the C21 ester [15,16].

### Stability of FF in freshly isolated human lung tissue

**Figure 3:**
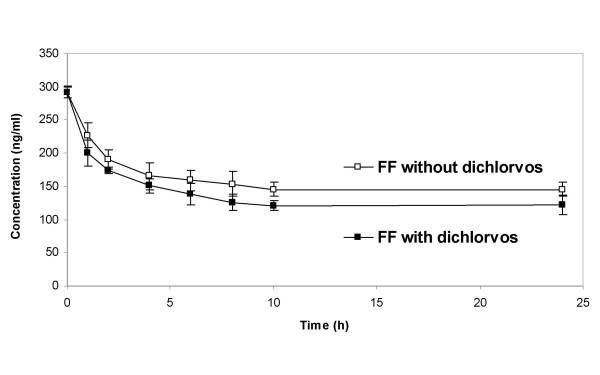
Stability of fluticasone furoate (FF) in human lung tissue suspensions of 37°C over 24 hours. Symbols represent the mean and mean deviation of the mean of four independent series of experiments. One incubation mixture contained the esterase inhibitor dichlorvos to determine a potential esterase mediated decomposition of the parent compound. No statistically significant differences between FF concentrations in the presence or absence of dichlorvos were observed at any of the analysed time points.

The stability of FF in the presence in human lung tissue was monitored over a period of 24 hours at an incubation temperature of 37°C (Figure 3). The incubations were performed in the presence and absence of the esterase inhibitor diclorvos. Indications of instability of the compound are either decreased compound concentrations in the supernatant in the absence of dichlorvos and/or the appearance of new peaks in the HPLC chromatograms. The initial decrease of FF concentration indicated the binding to the lung tissue pieces. Over the incubation period, concentrations of FF in the tissue supernatant were slightly higher in the absence of dichlorvos. No new peaks were observed in the HPLC chromatograms. No statistically significant differences were revealed between concentrations of FF in the presence and absence of the esterase inhibitor diclorvos at any of the single time points. Thus, non-specific esterase-catalyzed hydrolysis of FF did not occur in the presence of human lung tissue.

The enzymatic integrity of the lung tissue was demonstrated in a simultaneously performed control experiment with beclomethasone-17,21-dipropionate (BDP). The results of these control experiments were identical to those described previously [3]. In the absence of dichlorvos BDP concentrations in the supernatant rapidly decreased and the main metabolite beclomethasone-17-monopropionate (17-BMP) was detectable at high concentrations. Dichlorvos inhibited the decomposition of BDP and delayed the formation of 17-BMP up to 10 hours of incubation (data not shown).

### Lung tissue binding affinity of fluticasone furoate (FF)

**Figure 4:**
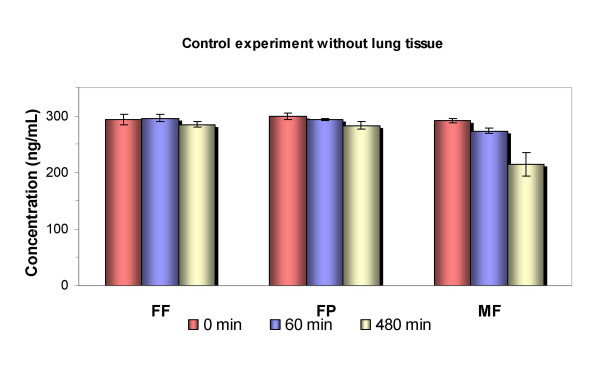
Control experiment for non-specific adsorption of glucocorticoids to incubation vials. The respective compounds were incubated in glass vials over 480 min at 37°C. The concentration in the supernatant was monitored. The decrease in concentrations of mometasone furoate (MF) indicated the degradation process of the compound. No adsorption was seen for fluticasone propionate (FP) and fluticasone furoate (FF). The columns represent the mean and mean deviation of the mean from triplicate experiments.

The binding affinity of FF in comparison with MF and FP to human lung tissue was determined in separate adsorption and desorption experiments. Control experiments for non-specific binding to incubation vials were performed in parallel with the respective glucocorticoid-containing buffer solutions under identical conditions. These control experiments revealed no non-specific binding of FF or FP to the glass incubation vials (Figure 4). A decrease in MF concentrations over 480 min at 37°C was paralleled by formation of the degradation product 9,11-epoxy MF as described earlier [3]. Thus, MF did not display non-specific binding to glass, but did show chemical instability.

**Figure 5:**
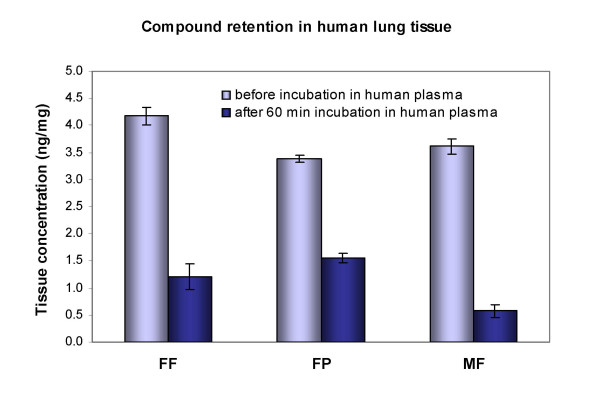
Comparison of concentrations of fluticasone furoate (FF), fluticasone propionate (FP) and mometasone furoate (MF) in human lung tissue. The columns represent the mean and mean deviation of the mean from four independent experiments. The left columns represent the compound concentration in tissue before incubation in human plasma. This tissue binding of FF was statistically significantly higher compared to FP (p ≤ 0.001) and MF (p ≤ 0.01). The right columns display the glucocorticoid concentrations remaining in the lung tissue after one hour equilibration with human plasma at 37°C. Remaining concentrations of FP and FF were not different while statistically significantly lower concentrations of MF were retained in the tissue compared to FF (p ≤ 0.01) and FP (p ≤ 0.001).

Adsorption of FF to human lung tissue *in vitro *occurred rapidly and was complete after about 20 min (data not shown). After 60 min incubation with the glucocorticoid-containing buffer at 37°C highest tissue binding was seen for FF (4.18 ± 0.16 ng/mg) and this was statistically significantly higher compared to FP (3.39 ± 0.06 ng/mg; p ≤ 0.001) and MF (3.65 ± 0.15 ng/mg; p ≤ 0.01) (Figure 5, left columns). FF also showed greater binding to human nasal tissue compared with FP in a single experiment with tissue pooled from 3 donors (data not shown).

The desorption of the glucocorticoids from lung tissue into human plasma revealed differences between the compounds (Figure 5, right columns). After 60 min highest concentrations of FP (1.55 ± 0.13 ng/mg) and FF (1.21 ± 0.23 ng/mg) were still present in the tissue. Remaining concentrations of FP and FF were not statistically significantly different. As reported previously [3], MF was rapidly redistributed from the lung tissue into human plasma and consequently lowest concentrations of mometasone furoate were detected in the tissue (0.57 ± 0.15 ng/mg). This was statistically significantly lower compared to FF (p ≤ 0.01) and FP (p ≤ 0.001).

## Discussion

Fluticasone furoate (FF) is a newly developed glucocorticoid for topical application. In the present investigation we characterized the receptor binding kinetics and the binding affinity to human lung tissue of FF in comparison with other latest generation glucocorticoids. We found that FF exhibited the highest ever described relative receptor affinity (RRA) of a topical glucocorticoid. The RRA of FF (2989 ± 135) exceeds the receptor affinities of all currently used corticosteroids such as mometasone furoate (MF; RRA = 2244 ± 142), fluticasone propionate (FP; RRA = 1775 ± 130), the active beclomethasone-17,21-dipropionate (BDP) metabolite beclomethasone-17-monopropionate (17-BMP; RRA = 1345 ± 125), ciclesonide's active principle (des-Cicle; RRA = 1212, *rat *receptor data) and budesonide (RRA = 855). Together with the compound's high retention in human lung tissue FF incorporates attributes that are suitable for topical anti-inflammatory therapy.

The substitution pattern of the steroidal D-ring is important for the affinity to the glucocorticoid receptor as well as for receptor selectivity [17]. For example, the D-ring substitution confers on MF highly potent glucocorticoid receptor binding affinity [4,18]. We deduced that D-ring modifications of MF were so favourable for high affinity binding to the glucocorticoid receptor that metabolic hydroxylation at the 6β position or loss of chlorine at the 9 position did not result in complete loss of ligand-binding properties.

One characteristic of the MF D-ring substitution pattern, the furoate moiety, is also present in FF. Consistent with the notion that the esterification of the 17α-OH by furoylation augments affinity we a found remarkably high RRA for FF that exceeds the RRA of e.g. FP by more than 60 %. This result is supported by recent X-ray crystal structure data of FF co-crystallized with the glucocorticoid receptor [19]. These data show the 17α-furoate ester fully occupying the lipophilic 17α pocket in the receptor and additional interactions with the receptor involving the 3-keto, 11β-hydroxy and 17β-fluoromethylthioester groups of the fluticasone backbone.

We determined the affinity of FF to the human lung glucocorticoid receptor by separate analysis of the receptor association and dissociation kinetics. This method is more precise compared to competition assays, especially for high affinity glucocorticoids [1]. For FF we observed a very fast and extensive association with the receptor, with an association rate constant significantly higher than for any other glucocorticoid. In contrast, the dissociation rate constant was almost identical to that of FP. Thus, the difference between FF and FP is mainly based on the more rapid and preferential binding of FF to the receptor. This kinetic behaviour of FF confirms our previous insights into receptor binding characteristics of high-affinity glucocorticoids [1,3]. FP was the first glucocorticoid with receptor binding clearly distinct from other compounds. In comparison with other glucocorticoids it displayed both a more rapid association and prolonged dissociation from the receptor [1]. The receptor binding kinetics of MF disclosed a high association rate constant while its dissociation rate was almost comparable to FP [3]. Thus, further increase in receptor affinity for FF was related to an increase in the association rate constant which is now also established for this compound.

Interestingly, those glucocorticoids with the highest RRAs do not comply with the previously described linear relationship between lipophilicity and receptor affinity [5]. FF, MF and FP reveal clear differences in their RRA, but their lipophilicity expressed as their relative retention times at a reversed-phase HPLC column is less different than their receptor affinities. However, the correlation between lipophilicity of the active compound and its RRA is still highly significant, though not linear, if the high affinity glucocorticoids FF, FP and MF are included into the analysis. There are glucocorticoids with higher lipophilicity such as BDP and Cicle, but these compounds are pro-drugs with virtually no affinity to the receptor. Both drugs gain activity by ester cleavage in C21 position. Thereby, however, they lose their high lipophilicity.

Since FF is not a pro-drug, its receptor binding affinity and thus activity is associated with the entire molecule. The compound is expected to be stable in the therapeutic target tissue. This is not necessarily seen for all glucocorticoids. We and other research groups recently observed that MF is not stable in lung tissue or plasma and undergoes chemical degradation [3,20,21]. We now elucidated the stability of FF in human lung tissue and found no degradation or metabolism within 24 h at 37°C. The esterase inhibitor dichlorvos was included in one of the incubation mixtures in case of an enzyme-catalyzed hydrolysis of the 17α furoate moiety or of the 17β S-fluoromethyl-carbothioate group. Neither did we determine the resulting metabolites or any other new peaks in the HPLC chromatograms nor did we observe lower FF concentrations in the tissue supernatant in the absence of dichlorvos. In the contrary, we found lower FF concentrations in the presence of the esterase inhibitor, though we do not have a clear explanation for this phenomenon. We conclude that there is no indication of instability or chemical modification of FF in the presence of enzymatically active human lung tissue.

Besides a high receptor binding affinity, a prolonged retention of the glucocorticoid in the lung tissue is a desired property. We compared the tissue binding behaviour of FF with FP and MF. After one hour equilibration of glucocorticoid-saturated lung tissue pieces with human plasma at 37°C, we found highest concentrations of FF and FP compared to MF remaining in the tissue. Obviously, these compounds have the most favourable tissue affinity and it should be expected that the distribution of these glucocorticoids from lung tissue into systemic circulation is slow *in vivo*. Clinical data confirm this for FP [22].

To conclude, we have characterized the novel glucocorticoid fluticasone furoate. Its relative receptor binding affinity exceeds the RRAs of all other currently clinically used glucocorticoids. Based on the tissue binding experiments a high retention of fluticasone furoate in human lung tissue is expected. These advantageous binding attributes may contribute to a highly efficacious profile for FF as a topical treatment for inflammatory disorders of the respiratory tract.

## Competing interests

Parts of this study were supported by a research grant of GlaxoSmithKline. This funding had no role in the collection, analysis and interpretation of data or in the writing of the manuscript.

## Authors' contributions

A.V. designed, carried out and analysed all the experiments and contributed to writing the manuscript.

P.H. conceived of the study, participated in the study design, performed the statistical analysis and drafted the manuscript.

All authors read and approved the final manuscript.
